# Systemic GDF11 stimulates the secretion of adiponectin and induces a calorie restriction‐like phenotype in aged mice

**DOI:** 10.1111/acel.13038

**Published:** 2019-10-22

**Authors:** Lida Katsimpardi, Nicolas Kuperwasser, Claire Camus, Carine Moigneu, Aurélie Chiche, Virginie Tolle, Han Li, Erzsebet Kokovay, Pierre‐Marie Lledo

**Affiliations:** ^1^ Perception and Memory Lab Neuroscience Department Institut Pasteur Paris France; ^2^ Centre National de la Recherche Scientifique Unité Mixte de Recherche 3571 Paris France; ^3^ Department of Cell Growth and Signaling Institut National de la Santé et de la Recherche Médicale (INSERM) U1151 Institut Necker Enfants Malades (INEM) Université Paris Descartes France; ^4^ Centre de Psychiatrie et Neurosciences UMR‐S 894 INSERM Université Paris Descartes Sorbonne Paris Cité Paris France; ^5^ Department of Developmental & Stem Cell Biology Cellular Plasticity & Disease Modelling CNRS UMR 3738 Institut Pasteur Paris France; ^6^ Cell Systems and Anatomy Brashop Institute for Longevity and Aging Studies University of Texas Health Science Center at San Antonio San Antonio TX USA

**Keywords:** adiponectin, aging, calorie restriction, GDF11, heterochronic parabiosis, rejuvenation

## Abstract

Aging is a negative regulator of general homeostasis, tissue function, and regeneration. Changes in organismal energy levels and physiology, through systemic manipulations such as calorie restriction and young blood infusion, can regenerate tissue activity and increase lifespan in aged mice. However, whether these two systemic manipulations could be linked has never been investigated. Here, we report that systemic GDF11 triggers a calorie restriction‐like phenotype without affecting appetite or GDF15 levels in the blood, restores the insulin/IGF‐1 signaling pathway, and stimulates adiponectin secretion from white adipose tissue by direct action on adipocytes, while repairing neurogenesis in the aged brain. These findings suggest that GDF11 has a pleiotropic effect on an organismal level and that it could be a linking mechanism of rejuvenation between heterochronic parabiosis and calorie restriction. As such, GDF11 could be considered as an important therapeutic candidate for age‐related neurodegenerative and metabolic disorders.

## Graphical abstract

GDF11 stimulates adipocytes to release adiponectin in the bloodstream of aged mice and induces a calorie restriction‐like phenotype without affecting appetite. At the same time, these same GDF11‐treated aged mice exhibit enhanced neurogenesis. This shows that GDF11 has pleiotropic effects in both the brain and the periphery of aged mice, linking the beneficial effects of both heterochronic parabiosis and calorie restriction.

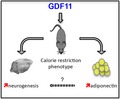

## 1 INTRODUCTION

Aging negatively affects organismal functions, including metabolic and homeostatic regulation, organ regeneration, and stem cell function, resulting in the progressive loss of the individual's capacity to self‐sustain (Brett & Rando, 2014). Each organ deteriorates at a different rate, and changes in one tissue are translated into organismal‐level alterations, through humoral factors, suggesting an extensive crosstalk between physiological actors (Zhang, Chen, & Liu, 2015). As such, aging is the most important risk factor for a multitude of diseases, such as neurodegeneration, metabolic disorders, cardiovascular disease, and cancer (Niccoli & Partridge, 2012).

However, it is possible to reverse or delay aging with genetic or systemic manipulations (Mahmoudi, Xu, & Brunet, 2019). Dietary interventions, such as intermittent fasting and calorie restriction (CR), are to date the most efficient ways to delay aging and increase lifespan across different species (Brandhorst et al., 2015; Colman et al., 2009). Because of their efficiency, many efforts are focused on finding molecules that mimic the effects of CR.

Intermittent fasting or fasting‐mimicking diets (FMDs) promote multi‐tissue regeneration, enhance cognitive performance, and extend healthspan in mice (Brandhorst et al., 2015). In humans, FMD beneficially affects subjects who were at risk for metabolic diseases (Wei et al., 2017). CR, a more long‐term regimen, consists of a 20%–40% reduction in calorie intake without malnutrition and is the most well‐studied dietary intervention (McCay, Crowell, & Maynard, 1935). CR induces an extension of lifespan of up to 50% in several organisms, including worms, rodents, and monkeys (Bordone & Guarente, 2005). A reduction in calorie intake prevents genetic changes and reduces the incidence of several diseases, such as cardiovascular disease, age‐associated cancer, and immune deficiencies, while increasing neurogenesis in the brain (Hursting, Perkins, Phang, & Barrett, 2001; Lane, Ingram, & Roth, 1999; Lee, Klopp, Weindruch, & Prolla, 1999; Lee, Seroogy, & Mattson, 2002; Mattson, 2010). Some of the physiological changes occurring in CR are believed to affect the insulin/IGF‐1 axis of aging. CR decreases serum IGF‐1 concentrations by 40% in rodents, and decreased IGF‐1 signaling is thought to be involved in delayed aging (Bonkowski, Rocha, Masternak, Al Regaiey, & Bartke, 2006; Dunn et al., 1997; Holzenberger et al., 2003). Another hormone involved in this process is adiponectin, which is secreted in response to CR and negative energy balance and its serum levels increase in mice subjected to CR (Combs et al., 2003). Adiponectin is a hormone with broad beneficial effects for the organism. It promotes antidiabetic effects by promoting insulin sensitivity (Combs et al., 2004; Maeda et al., 2002; Pajvani & Scherer, 2003; Yamauchi et al., 2003) and prevents atherosclerosis by attenuating chronic inflammation (Ohashi, Ouchi, & Matsuzawa, 2012; Okamoto et al., 2002; Yamamoto et al., 2005). Importantly, increased adiponectin levels are also associated with decreased growth hormone signaling and extended longevity in mice (Berryman et al., 2004; Otabe et al., 2007). Therefore, CR induces vast changes in the levels of circulating hormones, resulting in an altered composition of the systemic milieu.

Interestingly, youthful alterations of the systemic milieu have been recently shown to be crucial in rejuvenating multiple organs. Infusion of young factors in the aged blood changes the composition of the systemic milieu, via heterochronic parabiosis or injections of young plasma in aged mice. These methods have been very successful in rejuvenating several tissues, including those with low regenerative potential such as the heart, muscle, and central nervous system, as we and others have previously shown (Castellano et al., 2017; Conboy et al., 2005; Katsimpardi et al., 2014; Loffredo et al., 2013; Ruckh et al., 2012; Sinha et al., 2014; Villeda et al., 2011, 2014), suggesting that aging is a malleable process and that achieving the right systemic “cocktail” can activate tissue plasticity at almost any age.

The fact that altered blood composition by either heterochronic parabiosis or CR leads to organ rejuvenation, despite very different biological contexts, raises the exciting possibility that these two systemic manipulations may share common pathways and mechanisms, and that rejuvenation via parabiosis could be due, at least in part, to youthful factors acting as CR mimetics in the aged organism. One of the factors identified in parabiosis experiments was GDF11, which was shown to rejuvenate the aged brain (Katsimpardi et al., 2014), while having a broader rejuvenating effect on other peripheral aged organs, such as the muscle and heart (Loffredo et al., 2013; Sinha et al., 2014). Subsequently, other reports argued that GDF11 was positively associated with aging, cachexia, and inhibition of muscle regeneration in aged mice (Egerman et al., 2015; Harper et al., 2016; Jones et al., 2018), while different studies showed a beneficial role for GDF11 in the periphery (Poggioli et al., 2016; Su et al., 2019; Walker et al., 2016). In the central nervous system, GDF11 was also shown to be neuroprotective for neurovascular recovery and neurogenesis (Anqi, Ruiqi, Yanming, & Chao, 2019; Ma et al., 2018; Ozek, Krolewski, Buchanan, & Rubin, 2018; Schafer & LeBrasseur, 2019; Zhang et al., 2018).

While systemic administration of recombinant GDF11 protein induced a rejuvenating effect on the brain by increasing neurogenesis and vascular remodeling, GDF11‐injected aged mice also became lean (Katsimpardi et al., 2014; Ozek et al., 2018; Poggioli et al., 2016). This led us to hypothesize that the rejuvenation effect of GDF11 may result from a concerted action of this molecule on whole‐organismal physiology and that this interesting protein may be a rejuvenation mechanism coupling CR and heterochronic parabiosis.

## 2 RESULTS

**Figure 1:**
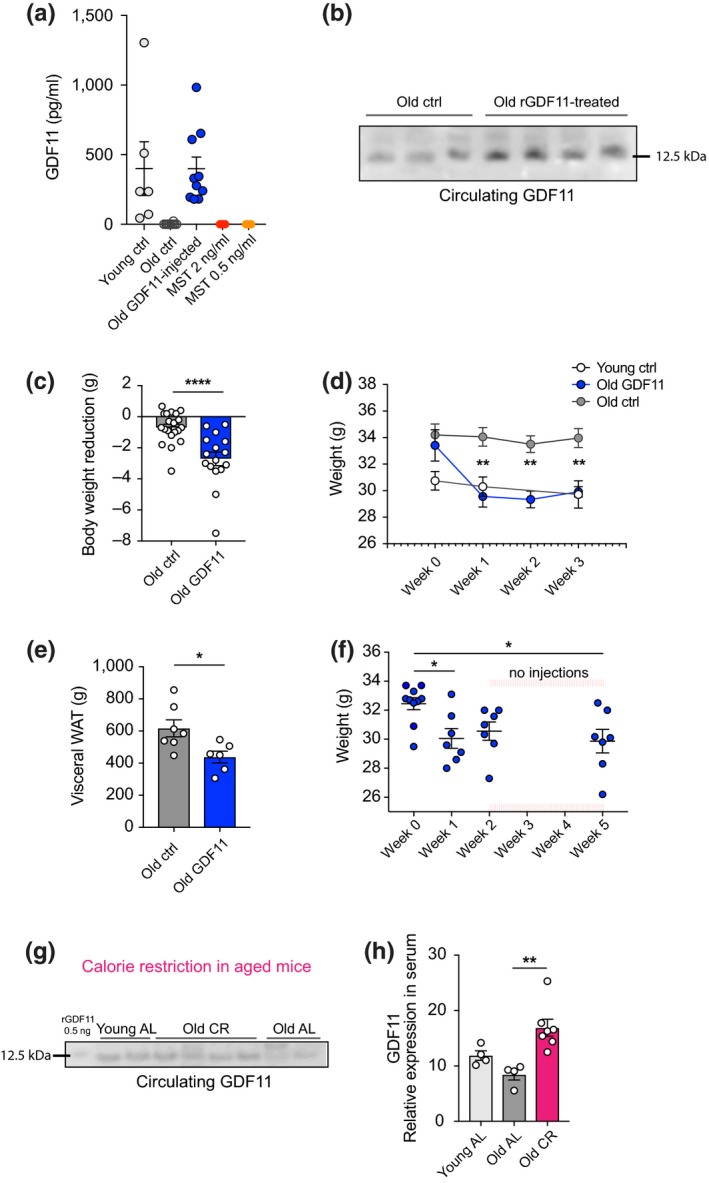
Serum GDF11 levels correlate with weight loss and calorie restriction. (a) ELISA measurements of circulating GDF11 in the plasma of young, old, and GDF11‐treated old mice 12 hr after injection (*n*_Y_ = 6, *n*_O_ = 10, *n*_GDF11_ = 10 mice per group). Recombinant MST (2 and 0.5 ng/ml) was used as a specificity control. (b) Western blot of equal serum volumes from aged GDF11‐injected and aged control mice probed with a specific anti‐GDF11 antibody. (c) Graphic representation of body weight reduction after 8 days of daily systemic GDF11 or saline administration in 22‐month‐old mice (*n* = 20 mice per group). (d) Weekly measurement of weight over 3 weeks of daily GDF11 or saline administration (*n* = 7 mice per group). (e) Measurement of WAT weight after 22 days of treatment (*n*_O_ = 7, *n*_GDF11_ = 6 mice per group). (f) Weekly weight measurement of mice injected with GDF11 for 2 weeks and monitored for 3 weeks without injections (*n* = 10 mice per group). (G) Western blot from equal volumes of young and old AL and old CR mice plasma probed with anti‐GDF11 antibody. (h) Quantification of (G) by optical intensity (*n*_Y‐AL_ = 4, *n*_O‐AL_ = 4, *n*_O‐CR_ = 7 mice per group). One‐way and two‐way ANOVA and Tukey's *post hoc* test for multiple group comparisons; Mann–Whitney test for two‐group comparisons; *p < .05, **p < .01; ****p < .0001; mean ± SEM

We sought to investigate the effect of systemic GDF11 treatment on organismal physiology and metabolism via daily intraperitoneal (IP) injections. First, we determined the levels of circulating GDF11 in the bloodstream after injection. Aged (22‐month‐old) mice were injected with recombinant GDF11 (rGDF11, 1 mg/kg) whereas control young (3‐ to 4‐month‐old) and aged (22‐month‐old) mice were injected with saline. Blood GDF11 was measured by sandwich ELISA 12 hr after the last injection. The average value of blood GDF11 in young mice was at 400 pg/ml. Aged GDF11‐injected mice presented an average value of blood GDF11 at 399 pg/ml, whereas the intrinsic circulating protein could not be detected in the blood of control aged mice (Figure 1a). Moreover, we confirmed the specificity of this assay for GDF11 by using recombinant myostatin (rMST), which was not detected at any concentration (Figure 1a and online detailed Materials and Methods). These results show that supplementation of rGDF11 injected IP at 1 mg/kg in aged mice increased the levels of blood GDF11 compared to intrinsic GDF11 circulating in the blood of control aged mice, bringing them to a youthful level. We repeated this measurement by Western blotting where the anti‐GDF11 antibody was fully validated for sensitivity and specificity to the GDF11 antigen (Figure S1 and online detailed Materials and Methods). Western blot analysis of GDF11‐injected or saline‐injected aged mice confirmed that GDF11‐injected mice exhibited significantly higher concentration of GDF11 in the blood compared to control aged mice (Figure 1b).

Next, we examined the effect of systemic GDF11 injections on body weight. Mice were IP injected daily at 7 p.m. in order to ensure that the protein is present during the active phase of mice (night time), and all injected mice were weighed weekly. After one week of daily administration, GDF11‐treated mice were significantly leaner than age‐matched controls (Figure 1c and Figure S2a), with an average reduction of 8% of their initial body weight (Figure S2b). Interestingly, as we continued the administration of GDF11, we observed no further weight loss after this time point following an additional two weeks of GDF11 treatment, and GDF11‐treated aged mice remained as lean as young mice and maintained a statistically significant weight difference compared to control aged mice for the rest of the treatment (Figure 1d). Analysis of the weight loss phenotype showed a strong reverse correlation between the degree of weight loss and the initial body weight in GDF11‐treated animals (Pearson's correlation coefficient, *r*_GDF11_ = −.739; Figure S2c). To examine their physical performance and motor coordination, we performed the rotarod test, where all aged mice were equally active and showed no signs of frailty at the end of the 3‐week GDF11 treatment (Figure S2d). We then examined the fat and muscle tissues of these mice. Visceral (epididymal) white adipose tissue (WAT) was significantly reduced after 3 weeks of treatment (Figure 1e), whereas tibia muscle mass remained the same (Figure S2e). Moreover, muscle sections from these mice were histologically analyzed by H&E staining, and no morphological changes were observed between the two aged groups (Figure S2f). Because of our previous finding that GDF11 can rejuvenate the aged brain, we also examined brain sections of the aged mice after 3 weeks of GDF11 or saline treatment. Quantification of doublecortin (DCX, a marker of migrating neuroblasts and neurogenesis) in the aged subventricular zone neurogenic niche revealed that these mice, which lost weight, also increased their neurogenic capacity (Figure S3), suggesting a simultaneous role for GDF11 in both brain rejuvenation and weight loss in aged mice.

Next, we asked whether the weight loss changes observed with GDF11 treatment would have a long‐lasting effect. Thus, we injected the aged mice with rGDF11 (1 mg/kg) or saline for two weeks to repeat the “weight loss and stabilization” period and then stopped injecting the mice. Indeed, GDF11‐treated mice lost weight the first week and subsequently reached a plateau. Interestingly, 3 weeks after we stopped the injections, these mice maintained the same weight, which was significantly reduced compared to the time point before the injections, despite the lack of GDF11 supplementation (Figure 1f). These findings suggest that systemic GDF11 administration triggers changes in organismal physiology that have a long‐lasting effect.

Since GDF11 seems to be directly associated with energy expenditure at the systemic level, we wondered how intrinsic GDF11 levels would be affected in a metabolically altered context. To answer this question, we performed CR in aged mice (22‐ to 28‐month‐old) and compared them to age‐matched ad libitum (AL)‐fed aged mice, as well as young (3.5‐month‐old) AL mice. Analysis of their serum by immunoblotting with the specific anti‐GDF11 antibody showed that levels of circulating GDF11 were increased in CR aged mice compared to their AL‐fed aged counterparts (Figure 1g,h). This finding provides further evidence that GDF11 levels in the blood correlate with organismal energy levels.

**Figure 2:**
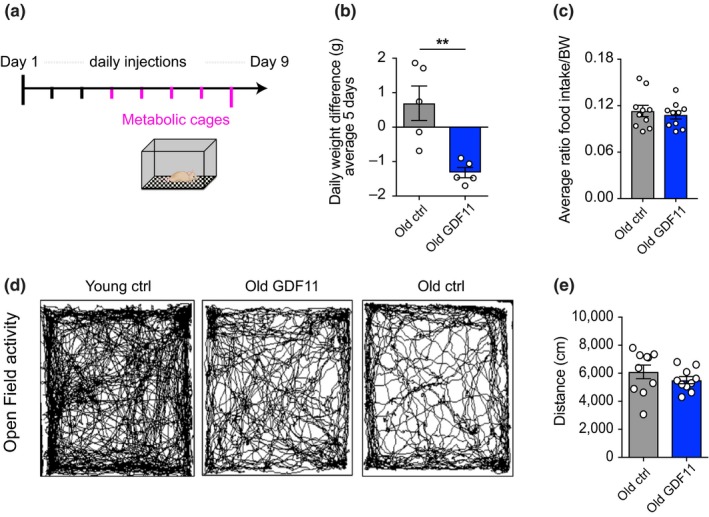
Food consumption and physical activity are not affected when GDF11‐treated aged mice lose weight. (a) Schematic representation of metabolic cage experiment: Young and aged mice were injected with saline or GDF11 for 3 days before transfer to metabolic cages, where they remained for 5 days while continuing to be injected daily. (b) Graphic representation of daily weight loss, averaged over 5 days (*n* = 5 mice per group). (c) Graphic representation of the average ratio of food intake per body weight (*n*_Y_ = 4, *n*_O_ = 10, *n*_GDF_ = 10 mice per group). (d) Measurement of distance travelled during 20 min in the open‐field arena (*n* = 9 mice per group). (g) Representative traces of mouse movements during 20 min of the open field test. Mann–Whitney test for two‐group comparisons; **p < .01; mean ± SEM

In order to better understand the effect of GDF11 on body weight changes, we decided to narrow our analysis to the specific window of the first week where weight loss was observed. In this window, we sought to investigate whether body weight reduction after systemic GDF11 administration was due to changes in appetite and food consumption. To address this question, young and aged mice were placed in metabolic cages on the third day of GDF11 or saline treatment for a period of 5 days (Figure 2a). While GDF11‐treated aged animals lost weight (Figure 2b), no changes in food intake were observed (Figure 2c). All aged mice traveled similar distances in the open‐field test (Figure 2d,e), suggesting that GDF11‐induced weight loss did not result from locomotor hyperactivity. Other metabolic and hormonal parameters, water consumption, urine and feces secretion, blood glucose, corticosterone, and leptin levels were examined. All of these parameters remained unchanged in GDF11‐treated aged mice compared to their controls (Figure S4d‐f, respectively). These findings demonstrate that systemic GDF11 induces weight loss without changes in appetite in aged mice.

**Figure 3:**
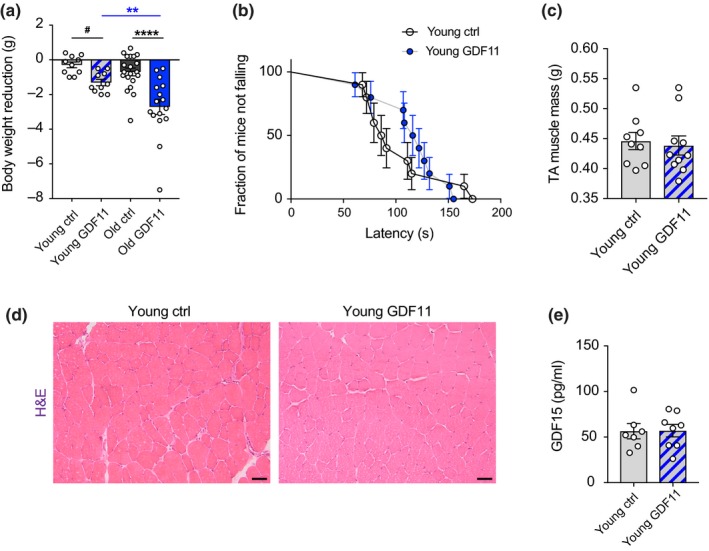
Young mice perform better and exhibit a moderate GDF15‐independent body weight reduction after systemic GDF11 treatment. (a) Graphic representation of body weight reduction after 8 days of daily systemic GDF11 or saline administration in young and aged mice (*n*_Y_ = 10, *n*_O_ = 20 mice per group). (b) Evaluation of young mice for their locomotor and coordination performance using the rotarod test. The survival curve represents the fraction of mice not falling from the rotarod over the latency to first fall. The data represent the best out of three trials for each mouse. (median_young‐ctrl_ = 55, median_young‐GDF11_ = 60; calculated using the Wilcoxon rank test). (c) Measurement of tibialis anterior muscle mass (*n*_Y_ = 9, *n*_YGDF11_ = 10 mice per group). (d) Histological analysis of tibialis anterior muscle sections by H&E staining. Scale bar: 50 μm. (e) ELISA measurements of circulating GDF15 in the plasma of saline or GDF11‐treated young mice (*n*_Y_ = 7, *n*_YGDF11_ = 8 mice per group). One‐way and two‐way ANOVA and Tukey's *post hoc* test for multiple group comparisons; Mann–Whitney test for two‐group comparisons; **p < .01; ****p < .0001; *#:* *t*‐test between young ctrl and young GDF11 mice with *p* = .0007; mean ± SEM

Since it was previously reported that supraphysiologic overexpression of GDF11 in the liver of young mice induced weight loss and cachexia through activation of GDF15 and anorexia (Jones et al., 2018), we sought to examine this in our paradigm of systemic rGDF11 administration. Thus, we injected 3‐month‐old young mice with GDF11 (1 mg/kg) IP for one week. In our paradigm, GDF11 induced a slight weight loss in young mice (4% reduction of their initial body weight), but significantly less than in old mice (Figure 3a). Interestingly, young GDF11‐treated mice showed enhanced performance in the rotarod test with a 5% increase in the median of the survival curve (median_young‐ctrl_ = 55, median_young‐GDF11_ = 60; Figure 3b). As in old mice, tibia muscle mass remained the same (Figure 3c) and no morphological changes were observed in muscle tissue after GDF11 administration (Figure 3d). Moreover, we examined GDF11‐injected and control young mice for GDF15 activation. We performed the previously reported ELISA assay for GDF15 detection (Jones et al., 2018) using the serum of these mice at one week after treatment, and saw no modification of GDF15 (Figure 3e). This demonstrates that a one‐week systemic GDF11 administration activates GDF15‐independent pathways for moderate body weight reduction in young mice.

**Figure 4:**
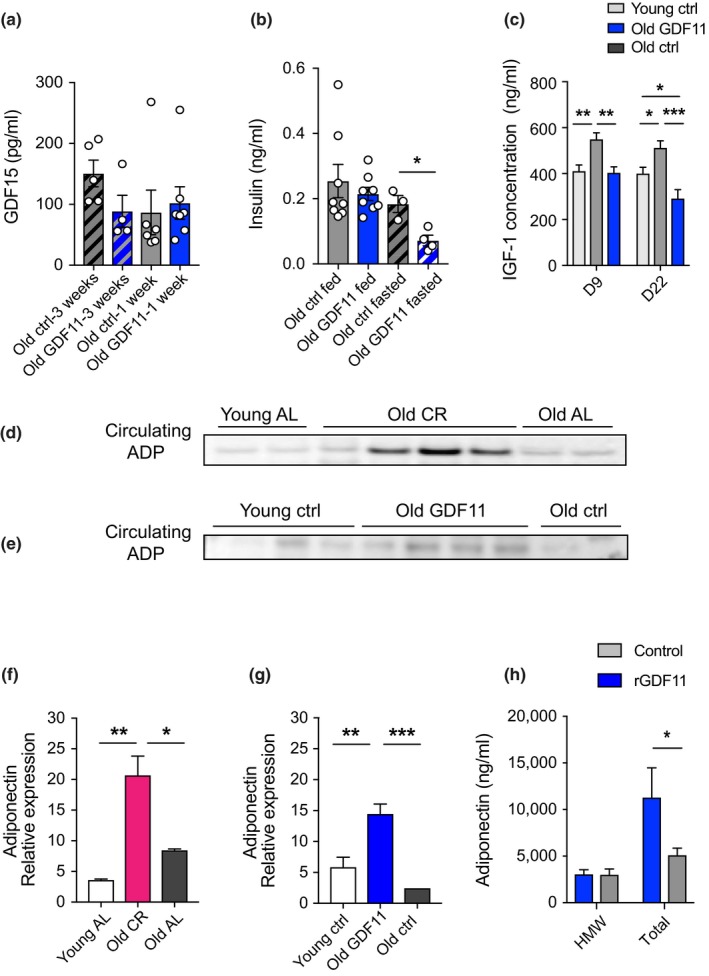
GDF11 treatment in aged mice induces hormonal changes similar to CR. (a) ELISA measurements of circulating GDF15 in the plasma of aged mice treated with either saline or GDF11 for 1 or 3 weeks (*n*_O‐3weeks_ = 5, *n*_GDF11‐3weeks_ = 4, *n*_O‐1week_ = 6, *n*_GDF11‐1week_ = 6 mice per group). (b) ELISA measurements of insulin in the plasma of fed or 6 hr‐fasted aged mice treated with either saline or GDF11 (*n*_O‐fed_ = 8, *n*_GDF11‐fed_ = 8, *n*_O‐fasted_ = 3, *n*_GDF11‐fasted_ = 4 mice per group). (C) ELISA measurement of circulating IGF‐1 in the plasma of young, old, and GDF11‐treated old mice (*n* = 5 mice per group). (d‐e) Representative Western blot images of equal volumes of serum from (d) young AL, old CR, and old AL and (e) young ctrl, old GDF11‐treated old ctrl, both probed with anti‐adiponectin antibody. (f) Quantification of (d) by optical density. (g) Quantification of (e) by optical density. (f) ELISA measurement of HMW or total adiponectin in the plasma of aged saline or 1‐week GDF11‐treated old mice (*n* = 4 mice per group). One‐way and two‐way ANOVA and Tukey's *post hoc* test for multiple group comparisons; Mann–Whitney test for two‐group comparisons; *p < .05, **p < .01; ***p < .001; mean ± SEM

Next, we sought to investigate which metabolic pathways were affected in aged mice after systemic GDF11 administration. Using the ELISA mentioned above, we measured GDF15 levels in serum of aged mice injected with GDF11 for either one week or for 3 weeks. In both cases, no significant change was observed, and there was even a trend for decreased GDF15 levels after 3 weeks of treatment (Figure 4a). Together with the fact that no anorexia was observed, these results suggest that GDF11 induces a healthy, GDF15‐independent weight loss in aged mice. We then sought to measure insulin levels, with or without fasting. For this, aged mice were treated with GDF11 or saline for 1 week, and then, mice were fed or fasted for 6 hr before blood collection. Insulin was measured in all samples using an ELISA. We found that aged GDF11‐treated mice that underwent fasting exhibited a significant decrease in insulin levels compared to control fasted mice, whereas no significant change was observed in fed mice (Figure 4b). In relation to this finding, we also measured plasma IGF‐1 levels, which were significantly decreased in old GDF11‐treated compared to old control mice, at both 9 and 22 days of treatment, showing that GDF11 rapidly induces metabolic changes that remain sustained over time (Figure 4c). Since weight loss was appetite‐independent but linked to a reduction in visceral WAT, we next examined adiponectin, an adipose‐secreted hormone that induces appetite‐independent weight loss (Qi et al., 2004). Adiponectin is inversely correlated with adipose mass and is known to increase in the context of CR (Combs et al., 2003). Indeed, in our CR paradigm, serum levels of adiponectin were increased solely in aged CR mice, the only mice that lost weight, compared to both young and old control AL mice (Figure 4d,f). Similarly, we observed elevated levels of adiponectin in aged GDF11‐treated mice, also the only group that underwent weight loss, compared to young and old control mice (Figure 4e,g). Adiponectin circulates in multiple isoforms in the blood, and total and high molecular weight (HMW) forms are inversely associated with obesity (Lubbers et al., 2013). In order to further examine which isoforms were affected in our paradigm, we performed an ELISA that distinguishes between these forms of adiponectin. This assay showed that total adiponectin was increased in the blood of GDF11‐treated mice, but no change was observed for the HMW isoform of circulating adiponectin (Figure 4h). Taken together, these results demonstrate that systemic GDF11 administration in aged mice is involved in metabolic pathways by inducing sustainable hormonal changes similar to those activated in CR.

**Figure 5:**
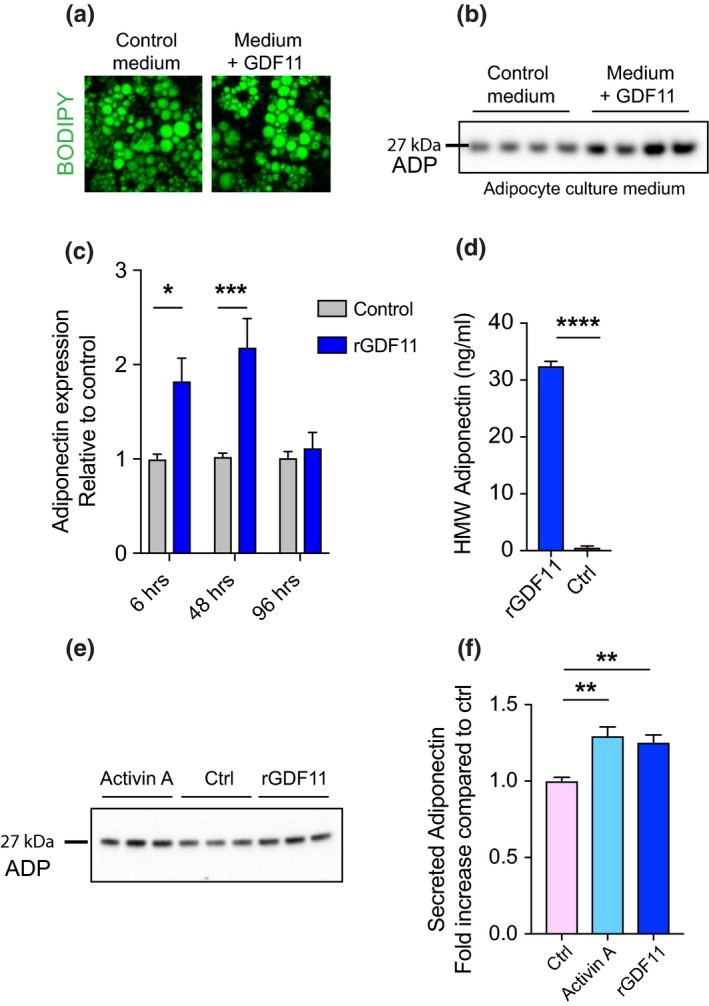
GDF11 stimulates adiponectin release in mature adipocyte cultures. (a) BODIPY staining of lipid droplets in mature adipocytes in vitro treated with rGDF11 or control medium. (b) Representative Western blot images of equal volumes of adipocyte culture medium after 6 hr of 20 ng/ml rGDF11 or no treatment probed with anti‐adiponectin antibody. (c) Quantification by Western blot of adiponectin release in adipocyte culture medium after 6, 48, and 96 hr of rGDF11 or no treatment (*n* = 4 conditioned medium samples per group). (d) ELISA measurement of HMW adiponectin in adipocyte conditioned medium after 6 hr of rGDF11 or no treatment (*n* = 3 conditioned medium samples per group). (e) Representative Western blot images of equal volumes of adipocyte culture medium after 6 hr of 20 ng/ml rGDF11, 20 ng/ml activin A or no treatment, probed with anti‐adiponectin antibody. (f) Quantification by Western blot of (E) (*n* = 6 conditioned medium samples per group). One‐way and two‐way ANOVA with Tukey's *post hoc* test for multiple group comparisons; Mann–Whitney test for two‐group comparisons; *p < .05, **p < .01, *** *p* < .001; ****p < .0001; mean ± SEM

Given the tight link between weight loss and adiponectin secretion, we wondered whether GDF11 might trigger this pathway by a direct action on white adipose tissue in aged mice; thus, we tested this hypothesis on mature, in vitro cultured, adipocytes. Adipocytes were differentiated from 3T3‐L1 cells, a pre‐adipocyte cell line, and were cultured until maturation, defined by the size of lipid droplets inside each cell by Oil Red O staining. Once adipocytes reached full maturation (large lipid droplets), they were treated with rGDF11 (20 ng/ml) in the medium for 6, 48, and 96 hr to establish a timeline of GDF11 action. Using BODIPY lipid staining, we observed no changes in morphology or lipid droplet size in mature adipocytes after GDF11 treatment at any time point (Figure 5a). Then, we measured adiponectin secretion over time into the culture medium by Western blotting (Figure 5b). Adiponectin levels were significantly increased compared to control medium after 6 hr and persisted up to 48 hr of GDF11 treatment (Figure 5c), indicating that GDF11 is efficient to quickly stimulate adiponectin secretion by adipocytes. Subsequently, we took advantage of a controlled context, the adipocyte culture, to examine whether GDF11 promotes secretion of HMW adiponectin. Using the ELISA mentioned above, we found that GDF11 significantly enhanced production of HMW adiponectin at 6 hr of treatment compared to the control medium (Figure 5d). Mechanistically, GDF11 signals downstream via the activin type‐IIA and type‐IIB receptors in most cell types (Oh et al., 2002; Walker et al., 2016). Therefore, we asked whether stimulation of these receptors by a different ligand would also enhance adiponectin secretion. Mature adipocytes were thus stimulated by Activin A (20 ng/ml) or GDF11 (20 ng/ml) for 6 hr. Western blot analysis of the conditioned medium showed an equally significant increase in adiponectin secretion by Activin A or GDF11 compared to the control condition (Figure 5f). These findings demonstrate that GDF11 acts directly on adipocytes to stimulate activin receptors in order to increase adiponectin release from adipocytes.

## 3 DISCUSSION

GDF11 is a protein with a variety of known roles in embryonic development, ranging from anteroposterior development to formation of multiple organs, including the central nervous system (Harmon et al., 2004; McPherron, Lawler, & Lee, 1997; Wu et al., 2003), yet its role in the aging organism has been controversial and its mechanism of action largely unknown.

Here, we present evidence that GDF11 induces a healthy calorie restriction‐like phenotype together with brain rejuvenation in aged mice, and it acts by stimulating the secretion of adiponectin directly on adipocytes. We demonstrate a potent role for GDF11 as a metabolic actor in the aged organism based on the following findings: (a) systemic administration of GDF11 induced healthy weight loss as early as 1 week after treatment, (b) this weight loss reached a plateau throughout the rest of the treatment and was maintained for 3 weeks beyond the end of the treatment, (c) GDF11 levels were increased in aged mice that were subjected to CR, (d) metabolic changes were independent of GDF15 activation or anorexia, but correlated with changes in adiponectin levels and the insulin/IGF‐1 metabolic pathway, (e) GDF11 activated adiponectin secretion directly from adipocytes, and (f) all the above changes correlated with a brain rejuvenation phenotype in aged mice.

The fact that systemic administration of GDF11 induced healthy weight loss as early as 1 week after treatment and subsequently reached a plateau comes to accordance with previous reports (Ozek et al., 2018; Poggioli et al., 2016). During and after the treatment, all mice were healthy and displayed no signs of cachexia or frailty contrary to previous reports (Egerman et al., 2015). GDF11 treatment did not affect food intake, appetite, or locomotor activity in aged mice. Moreover, muscle sections of treated aged mice showed no morphological or histological alterations after a 3‐week GDF11 treatment.

It was previously reported that young mice, where GDF11 was supraphysiologically expressed through plasmid insertion into the liver, lost weight due to anorexia and GDF15 activation, and displayed signs of frailty (Jones et al., 2018). In our work here, systemic GDF11 administration in young mice did not affect GDF15 levels in the blood, and GDF11‐treated mice exhibited an increased performance in the rotarod test. In fact, GDF15 levels remained unchanged regardless of the age of the mice or the length of GDF11 treatment. In addition, in the Jones et al. study, mice steadily lost weight, reaching a level of 35% reduction of their initial weight, whereas we found that mice only lost 4% of their initial weight after one week and then reached a plateau. In our paradigm, IP injections of 1 mg/kg rGDF11 resulted in an average blood concentration of GDF11 of 399 pg/ml, which suggests that injected rGDF11 only partially enters the bloodstream. In the Jones et al. (2018) study, the resulting concentration of GDF11 levels in the blood was reported to be over 3 μg/ml for the 3 μg plasmid insertion and over 12 μg/ml for the 10 μg plasmid insertion in the liver, thus the levels of circulating GDF11 in that study were between 7,500 to 30,000 times higher than in our GDF11‐injected mice. Therefore, a high, possibly toxic, dose of liver‐secreted GDF11 in the blood and/or the different methodology of GDF11 delivery could alter the functional state of the liver (and other systems) and induce the expression of GDF15.

Our findings propose a role for GDF11 as a molecule that is directly coupled with a CR‐like phenotype. Indeed, increased blood levels of GDF11 are correlated with body weight reduction, whether in the context of GDF11 treatment or in the context of CR. This is also corroborated by the fact that after the initial weight reduction, treated mice maintained a lower, youthful weight for another 3 weeks without any further GDF11 supplementation, suggesting that GDF11 induces secondary hormonal changes that are stable for a long period of time. Similarly, mice subjected to CR exhibit body weight reduction, improved insulin sensitivity, and lower cholesterol and blood pressure (Anderson, Shanmuganayagam, & Weindruch, 2009; Fontana, Meyer, Klein, & Holloszy, 2004). The insulin/IGF‐1 axis of aging is also affected in CR (Weindruch & Sohal, 1997) and IGF‐1 levels are inversely correlated with obesity and aging (Chaker, Aid, Berry, & Holzenberger, 2015; Fontana, Weiss, Villareal, Klein, & Holloszy, 2008; Holzenberger et al., 2003). GDF11 treatment led to a 27% reduction in serum levels of IGF‐1, in a sustainable manner throughout GDF11 administration, which represents a reduction similar to the effects of CR, together with a decrease in fasting insulin levels. Given that reduced levels of IGF‐1 and insulin are tightly linked to increased longevity (Holzenberger, 2011; Holzenberger et al., 2003), it would be interesting to examine whether longer GDF11 treatment could increase longevity.

Mechanistically, we provide evidence that GDF11 acts directly on adipocytes to induce adiponectin secretion. Adiponectin regulates energy expenditure by acting directly in the brain to reduce weight without affecting appetite (Qi et al., 2004), and its levels are known to increase in the context of CR (Miller et al., 2017), suggesting that this hormone may have an important role in regulating systemic energy levels in the GDF11 paradigm. It is interesting to note that adiponectin was equally affected in both GDF11 and CR paradigms, lending credence to the idea that GDF11 mimics CR effects. We also examined the different circulating forms of adiponectin. While some studies report a prevalence of HMW adiponectin for improvement of insulin sensitivity (Pajvani et al., 2004), other studies suggest that the two isoforms might exhibit different concentrations in the blood (Berryman et al., 2010). In our paradigm, total adiponectin was increased after GDF11 treatment, whereas HMW adiponectin remained unchanged in the serum of aged mice. These results could be explained by the fact that, unlike total adiponectin, HMW adiponectin levels vary with age (Lubbers et al., 2013). The finding that GDF11 treatment on adipocyte cultures greatly increased HMW secretion also corroborates this hypothesis.

Mechanistically, we show that adiponectin secretion was due to stimulation of activin receptors. However, it is still unknown whether all the metabolic effects of GDF11 are due to adiponectin activation or whether GDF11 could act simultaneously on other targets. Moreover, since adiponectin has multiple antidiabetic, anti‐atherogenic effects, it would be crucial to explore whether a synergistic administration of GDF11 and adiponectin would further enhance the beneficial effects of this treatment in age‐related and metabolic disorders.

Lastly, it is extremely important to note that the CR‐like phenotype induced by GDF11 correlated with a rejuvenation phenotype in the brain, suggesting that GDF11 treatment might reverse brain dysfunctions related to aging along with a pleiotropic effect on whole‐body metabolism. This could be partly due to a synergistic action of GDF11 with adiponectin, which also enhances neurogenesis and is thought to mediate the beneficial effects of exercise in the brain (Yau et al., 2014). Therefore, it would be interesting to further explore the mechanistic link between GDF11 and adiponectin on brain functions and whether knocking down adiponectin would reduce the beneficial effects of systemic GDF11 in the brain.

## 4 EXPERIMENTAL PROCEDURES

### 4.1 Animals

Young (3 months) and aged (22 months) C57BL/6JRj mice were obtained from Janvier Labs (France). For the calorie restriction study, male C57BL/6J mice were obtained from Jackson Laboratories. All animals were group housed (except for the metabolic cage experiment) and provided free access to water. All animal procedures were performed in accordance with French legislation and in compliance with the European Communities Council Directives (2010/63/UE), according to the regulations of Institut Pasteur Animal Care Committees. All calorie‐restricted (CR) and ad libitum (AL) mice studies were approved by the University of Texas Health Science Center at San Antonio Institutional Animal Care and Use Committee and performed in accordance with institutional and federal guidelines. All samples were collected from fed mice, except for the fasting experiment for the measurement of insulin levels.

### 4.2 Calorie restriction

Calories were restricted in a stepwise fashion to 40% of free feeding weight by 16 weeks of age (10% restriction at 14 weeks, 25% restriction at 15 weeks, and 40% restriction at 16 weeks). Age‐matched controls were fed ad libitum. Mice were maintained on this diet until sacrifice at 22–28 months of age. Young AL mice were 3.5‐month‐old male C57BL/6J. All animals were group housed and provided free access to water.

### 4.3 Metabolic cages

Young and old mice were placed in metabolic cages for 5 days, starting 3 days after the first injection of GDF11 or saline. Each metabolic cage accommodated 1 mouse. Metabolic cages assured the complete separation of urine and feces. Food and water consumption, as well as feces and urine secretion, was measured daily at the same time of day to take into account circadian influences. Mice were weighed daily, at the same time of the day. The metabolic cage experiment was repeated twice.

### 4.4 GDF11 administration

GDF11 (Peprotech, Cat# 120‐11) was dissolved in water, further diluted according to the manufacturer's instructions, and injected at a concentration of 1 mg/kg. Control mice (young or aged) were injected with equivalent volumes of saline. Injection concentration was chosen based on a pilot study with three different concentrations (0.1, 0.5, and 1.0 mg/kg) where consistent weight loss and brain rejuvenation were observed upon 1 mg/kg GDF11 administration. The half‐life of GDF11 at these concentrations was found to be of 12 hr.

## CONFLICT OF INTEREST

The authors declare no competing interests.

## AUTHOR CONTRIBUTIONS

L.K. conceptualized the project, designed and performed research, analyzed the data, and wrote the manuscript; N.K. performed research, provided suggestions for experiments, and analyzed the data; C.C., C.M., and A.C. performed research; V.T., H.L., and E.K. provided material; P.‐M.L. and L.K. supervised the study.

## References

[acel13038-bib-0001] Anderson, R. M. , Shanmuganayagam, D. , & Weindruch, R. (2009). Caloric restriction and aging: Studies in mice and monkeys. Toxicologic Pathology, 37, 47–51. 10.1177/0192623308329476 19075044 PMC3734859

[acel13038-bib-0002] Anqi, X. , Ruiqi, C. , Yanming, R. , & Chao, Y. (2019). Neuroprotective potential of GDF11 in experimental intracerebral hemorrhage in elderly rats. Journal of Clinical Neuroscience, 63, 182–188. 10.1016/j.jocn.2019.02.016 30827882

[acel13038-bib-0003] Berryman, D. E. , List, E. O. , Coschigano, K. T. , Behar, K. , Kim, J. K. , & Kopchick, J. J. (2004). Comparing adiposity profiles in three mouse models with altered GH signaling. Growth Hormone and IGF Research, 14, 309–318. 10.1016/j.ghir.2004.02.005 15231300

[acel13038-bib-0004] Berryman, D. E. , List, E. O. , Palmer, A. J. , Chung, M. Y. , Wright‐Piekarski, J. , Lubbers, E. , … Kopchick, J. J. (2010). Two‐year body composition analyses of long‐lived GHR null mice. Journals of Gerontology Series A: Biological Sciences and Medical Sciences, 65, 31–40. 10.1093/gerona/glp175 PMC2796884 19901018

[acel13038-bib-0005] Bonkowski, M. S. , Rocha, J. S. , Masternak, M. M. , Al Regaiey, K. A. , & Bartke, A. (2006). Targeted disruption of growth hormone receptor interferes with the beneficial actions of calorie restriction. Proceedings of the National Academy of Sciences of the United States of America, 103, 7901–7905. 10.1073/pnas.0600161103 16682650 PMC1458512

[acel13038-bib-0006] Bordone, L. , & Guarente, L. (2005). Calorie restriction, SIRT1 and metabolism: Understanding longevity. Nature Reviews Molecular Cell Biology, 6, 298–305. 10.1038/nrm1616 15768047

[acel13038-bib-0007] Brandhorst, S. , Choi, I. Y. , Wei, M. , Cheng, C. W. , Sedrakyan, S. , Navarrete, G. , … Longo, V. D. (2015). A periodic diet that mimics fasting promotes multi‐system regeneration, enhanced cognitive performance, and healthspan. Cell Metabolism, 22, 86–99. 10.1016/j.cmet.2015.05.012 26094889 PMC4509734

[acel13038-bib-0008] Brett, J. O. , & Rando, T. A. (2014). Alive and well? Exploring disease by studying lifespan. Current Opinion in Genetics & Development, 26, 33–40. 10.1016/j.gde.2014.05.004 25005743 PMC4253307

[acel13038-bib-0009] Castellano, J. M. , Mosher, K. I. , Abbey, R. J. , McBride, A. A. , James, M. L. , Berdnik, D. , … Wyss‐Coray, T. (2017). Human umbilical cord plasma proteins revitalize hippocampal function in aged mice. Nature, 544, 488–492. 10.1038/nature22067 28424512 PMC5586222

[acel13038-bib-0010] Chaker, Z. , Aid, S. , Berry, H. , & Holzenberger, M. (2015). Suppression of IGF‐I signals in neural stem cells enhances neurogenesis and olfactory function during aging. Aging Cell, 14, 847–856. 10.1111/acel.12365 26219530 PMC4568972

[acel13038-bib-0011] Colman, R. J. , Anderson, R. M. , Johnson, S. C. , Kastman, E. K. , Kosmatka, K. J. , Beasley, T. M. , … Weindruch, R. (2009). Caloric restriction delays disease onset and mortality in rhesus monkeys. Science, 325, 201–204. 10.1126/science.1173635 19590001 PMC2812811

[acel13038-bib-0012] Combs, T. P. , Berg, A. H. , Rajala, M. W. , Klebanov, S. , Iyengar, P. , Jimenez‐Chillaron, J. C. , … Scherer, P. E. (2003). Sexual differentiation, pregnancy, calorie restriction, and aging affect the adipocyte‐specific secretory protein adiponectin. Diabetes, 52, 268–276. 10.2337/diabetes.52.2.268 12540596

[acel13038-bib-0013] Combs, T. P. , Pajvani, U. B. , Berg, A. H. , Lin, Y. , Jelicks, L. A. , Laplante, M. , … Scherer, P. E. (2004). A transgenic mouse with a deletion in the collagenous domain of adiponectin displays elevated circulating adiponectin and improved insulin sensitivity. Endocrinology, 145, 367–383. 10.1210/en.2003-1068 14576179

[acel13038-bib-0014] Conboy, I. M. , Conboy, M. J. , Wagers, A. J. , Girma, E. R. , Weissman, I. L. , & Rando, T. A. (2005). Rejuvenation of aged progenitor cells by exposure to a young systemic environment. Nature, 433, 760–764. 10.1038/nature03260 15716955

[acel13038-bib-0015] Dunn, S. E. , Kari, F. W. , French, J. , Leininger, J. R. , Travlos, G. , Wilson, R. , & Barrett, J. C. (1997). Dietary restriction reduces insulin‐like growth factor I levels, which modulates apoptosis, cell proliferation, and tumor progression in p53‐deficient mice. Cancer Research, 57, 4667–4672. 9354418

[acel13038-bib-0016] Egerman, M. A. , Cadena, S. M. , Gilbert, J. A. , Meyer, A. , Nelson, H. N. , Swalley, S. E. , … Glass, D. J. (2015). GDF11 Increases with Age and Inhibits Skeletal Muscle Regeneration. Cell Metabolism, 22, 164–174. 10.1016/j.cmet.2015.05.010 26001423 PMC4497834

[acel13038-bib-0017] Fontana, L. , Meyer, T. E. , Klein, S. , & Holloszy, J. O. (2004). Long‐term calorie restriction is highly effective in reducing the risk for atherosclerosis in humans. Proceedings of the National Academy of Sciences of the United States of America, 101, 6659–6663. 10.1073/pnas.0308291101 15096581 PMC404101

[acel13038-bib-0018] Fontana, L. , Weiss, E. P. , Villareal, D. T. , Klein, S. , & Holloszy, J. O. (2008). Long‐term effects of calorie or protein restriction on serum IGF‐1 and IGFBP‐3 concentration in humans. Aging Cell, 7, 681–687. 10.1111/j.1474-9726.2008.00417.x 18843793 PMC2673798

[acel13038-bib-0019] Harmon, E. B. , Apelqvist, A. A. , Smart, N. G. , Gu, X. , Osborne, D. H. , & Kim, S. K. (2004). GDF11 modulates NGN3+ islet progenitor cell number and promotes beta‐cell differentiation in pancreas development. Development, 131, 6163–6174. 15548585 10.1242/dev.01535

[acel13038-bib-0020] Harper, S. C. , Brack, A. , MacDonnell, S. , Franti, M. , Olwin, B. B. , Bailey, B. A. , … Houser, S. R. (2016). Is growth differentiation factor 11 a realistic therapeutic for aging‐dependent muscle defects? Circulation Research, 118(7), 1143–1150. 27034276 10.1161/CIRCRESAHA.116.307962 PMC4829942

[acel13038-bib-0021] Holzenberger, M. (2011). Igf‐I signaling and effects on longevity. Nestle Nutrition workshop series Paediatric programme 68, 237–245; discussion 246–239. 10.1159/000325914 22044904

[acel13038-bib-0022] Holzenberger, M. , Dupont, J. , Ducos, B. , Leneuve, P. , Geloen, A. , Even, P. C. , … Le Bouc, Y. (2003). IGF‐1 receptor regulates lifespan and resistance to oxidative stress in mice. Nature, 421, 182–187. 10.1038/nature01298 12483226

[acel13038-bib-0023] Hursting, S. D. , Perkins, S. N. , Phang, J. M. , & Barrett, J. C. (2001). Diet and cancer prevention studies in p53‐deficient mice. Journal of Nutrition, 131, 3092S–3094S. 10.1093/jn/131.11.3092S 11694654

[acel13038-bib-0024] Jones, J. E. , Cadena, S. M. , Gong, C. , Wang, X. , Chen, Z. , Wang, S. X. , … Glass, D. J. (2018). Supraphysiologic administration of GDF11 induces cachexia in part by upregulating GDF15. Cell Reports, 22, 1522–1530. 10.1016/j.celrep.2018.01.044 29425507

[acel13038-bib-0025] Katsimpardi, L. , Litterman, N. K. , Schein, P. A. , Miller, C. M. , Loffredo, F. S. , Wojtkiewicz, G. R. , … Rubin, L. L. (2014). Vascular and neurogenic rejuvenation of the aging mouse brain by young systemic factors. Science, 344, 630–634. 10.1126/science.1251141 24797482 PMC4123747

[acel13038-bib-0026] Lane, M. A. , Ingram, D. K. , & Roth, G. S. (1999). Calorie restriction in nonhuman primates: Effects on diabetes and cardiovascular disease risk. Toxicological Sciences, 52, 41–48. 10.1093/toxsci/52.suppl_1.41 10630589

[acel13038-bib-0027] Lee, C. K. , Klopp, R. G. , Weindruch, R. , & Prolla, T. A. (1999). Gene expression profile of aging and its retardation by caloric restriction. Science, 285, 1390–1393. 10.1126/science.285.5432.1390 10464095

[acel13038-bib-0028] Lee, J. , Seroogy, K. B. , & Mattson, M. P. (2002). Dietary restriction enhances neurotrophin expression and neurogenesis in the hippocampus of adult mice. Journal of Neurochemistry, 80, 539–547. 10.1046/j.0022-3042.2001.00747.x 11905999

[acel13038-bib-0029] Loffredo, F. S. , Steinhauser, M. L. , Jay, S. M. , Gannon, J. , Pancoast, J. R. , Yalamanchi, P. , … Lee, R. T. (2013). Growth differentiation factor 11 is a circulating factor that reverses age‐related cardiac hypertrophy. Cell, 153, 828–839. 10.1016/j.cell.2013.04.015 23663781 PMC3677132

[acel13038-bib-0030] Lubbers, E. R. , List, E. O. , Jara, A. , Sackman‐Sala, L. , Cordoba‐Chacon, J. , Gahete, M. D. , … Berryman, D. E. (2013). Adiponectin in mice with altered GH action: Links to insulin sensitivity and longevity? Journal of Endocrinology, 216, 363–374. 10.1530/JOE-12-0505 23261955 PMC3756886

[acel13038-bib-0031] Ma, J. , Zhang, L. , Niu, T. , Ai, C. , Jia, G. , Jin, X. , … Li, C. (2018). Growth differentiation factor 11 improves neurobehavioral recovery and stimulates angiogenesis in rats subjected to cerebral ischemia/reperfusion. Brain Research Bulletin, 139, 38–47. 10.1016/j.brainresbull.2018.02.011 29432795

[acel13038-bib-0032] Maeda, N. , Shimomura, I. , Kishida, K. , Nishizawa, H. , Matsuda, M. , Nagaretani, H. , … Matsuzawa, Y. (2002). Diet‐induced insulin resistance in mice lacking adiponectin/ACRP30. Nature Medicine, 8, 731–737. 10.1038/nm724 12068289

[acel13038-bib-0033] Mahmoudi, S. , Xu, L. , & Brunet, A. (2019). Turning back time with emerging rejuvenation strategies. Nature Cell Biology, 21, 32–43. 10.1038/s41556-018-0206-0 30602763 PMC7653017

[acel13038-bib-0034] Mattson, M. P. (2010). The impact of dietary energy intake on cognitive aging. Frontiers in Aging Neuroscience, 2, 5 10.3389/neuro.24.005.2010 20552045 PMC2874403

[acel13038-bib-0035] McCay, C. M. , Crowell, M. F. , & Maynard, L. A. (1935). The effect of retarded growth upon the length of life span and upon the ultimate body size. Nutrition, 5, 155–171; discussion 172. 2520283

[acel13038-bib-0036] McPherron, A. C. , Lawler, A. M. , & Lee, S. J. (1997). Regulation of skeletal muscle mass in mice by a new TGF‐beta superfamily member. Nature, 387, 83–90. 9139826 10.1038/387083a0

[acel13038-bib-0037] Miller, K. N. , Burhans, M. S. , Clark, J. P. , Howell, P. R. , Polewski, M. A. , DeMuth, T. M. , … Anderson, R. M. (2017). Aging and caloric restriction impact adipose tissue, adiponectin, and circulating lipids. Aging Cell, 16, 497–507. 10.1111/acel.12575 28156058 PMC5418198

[acel13038-bib-0038] Niccoli, T. , & Partridge, L. (2012). Ageing as a risk factor for disease. Current Biology, 22, R741–752. 10.1016/j.cub.2012.07.024 22975005

[acel13038-bib-0039] Oh, S. P. , Yeo, C. Y. , Lee, Y. , Schrewe, H. , Whitman, M. , & Li, E. (2002). Activin type IIA and IIB receptors mediate Gdf11 signaling in axial vertebral patterning. Genes and Development, 16, 2749–2754. 10.1101/gad.1021802 12414726 PMC187472

[acel13038-bib-0040] Ohashi, K. , Ouchi, N. , & Matsuzawa, Y. (2012). Anti‐inflammatory and anti‐atherogenic properties of adiponectin. Biochimie, 94, 2137–2142. 10.1016/j.biochi.2012.06.008 22713764

[acel13038-bib-0041] Okamoto, Y. , Kihara, S. , Ouchi, N. , Nishida, M. , Arita, Y. , Kumada, M. , … Matsuzawa, Y. (2002). Adiponectin reduces atherosclerosis in apolipoprotein E‐deficient mice. Circulation, 106, 2767–2770. 10.1161/01.CIR.0000042707.50032.19 12451000

[acel13038-bib-0042] Otabe, S. , Yuan, X. , Fukutani, T. , Wada, N. , Hashinaga, T. , Nakayama, H. , … Yamada, K. (2007). Overexpression of human adiponectin in transgenic mice results in suppression of fat accumulation and prevention of premature death by high‐calorie diet. American Journal of Physiology‐Endocrinology and Metabolism, 293, E210–E218. 10.1152/ajpendo.00645.2006 17389708

[acel13038-bib-0043] Ozek, C. , Krolewski, R. C. , Buchanan, S. M. , & Rubin, L. L. (2018). Growth Differentiation Factor 11 treatment leads to neuronal and vascular improvements in the hippocampus of aged mice. Scientific Reports, 8, 17293 10.1038/s41598-018-35716-6 30470794 PMC6251885

[acel13038-bib-0044] Pajvani, U. B. , Hawkins, M. , Combs, T. P. , Rajala, M. W. , Doebber, T. , Berger, J. P. , … Scherer, P. E. (2004). Complex distribution, not absolute amount of adiponectin, correlates with thiazolidinedione‐mediated improvement in insulin sensitivity. The Journal of Biological Chemistry, 279, 12152–12162. 10.1074/jbc.M311113200 14699128

[acel13038-bib-0045] Pajvani, U. B. , & Scherer, P. E. (2003). Adiponectin: Systemic contributor to insulin sensitivity. Current Diabetes Reports, 3, 207–213. 10.1007/s11892-003-0065-2 12762967

[acel13038-bib-0046] Poggioli, T. , Vujic, A. , Yang, P. , Macias‐Trevino, C. , Uygur, A. , Loffredo, F. S. , … Lee, R. T. (2016). Circulating growth differentiation factor 11/8 levels decline with age. Circulation Research, 118, 29–37. 10.1161/CIRCRESAHA.115.307521 26489925 PMC4748736

[acel13038-bib-0047] Qi, Y. , Takahashi, N. , Hileman, S. M. , Patel, H. R. , Berg, A. H. , Pajvani, U. B. , … Ahima, R. S. (2004). Adiponectin acts in the brain to decrease body weight. Nature Medicine, 10, 524–529. 10.1038/nm1029 15077108

[acel13038-bib-0048] Ruckh, J. M. , Zhao, J. W. , Shadrach, J. L. , van Wijngaarden, P. , Rao, T. N. , Wagers, A. J. , & Franklin, R. J. (2012). Rejuvenation of regeneration in the aging central nervous system. Cell Stem Cell, 10, 96–103. 10.1016/j.stem.2011.11.019 22226359 PMC3714794

[acel13038-bib-0049] Schafer, M. J. , & LeBrasseur, N. K. (2019). The influence of GDF11 on brain fate and function. Geroscience, 41, 1–11. 10.1007/s11357-019-00054-6 30729414 PMC6423340

[acel13038-bib-0050] Sinha, M. , Jang, Y. C. , Oh, J. , Khong, D. , Wu, E. Y. , Manohar, R. , … Wagers, A. J. (2014). Restoring systemic GDF11 levels reverses age‐related dysfunction in mouse skeletal muscle. Science, 344, 649–652. 10.1126/science.1251152 24797481 PMC4104429

[acel13038-bib-0051] Su, H. H. , Liao, J. M. , Wang, Y. H. , Chen, K. M. , Lin, C. W. , Lee, I. H. , … Huang, S. S. (2019). Exogenous GDF11 attenuates non‐canonical TGF‐beta signaling to protect the heart from acute myocardial ischemia‐reperfusion injury. Basic Research in Cardiology, 114, 20. 30900023 10.1007/s00395-019-0728-z

[acel13038-bib-0052] Villeda, S. A. , Luo, J. , Mosher, K. I. , Zou, B. , Britschgi, M. , Bieri, G. , … Wyss‐Coray, T. (2011). The ageing systemic milieu negatively regulates neurogenesis and cognitive function. Nature, 477, 90–94. 10.1038/nature10357 21886162 PMC3170097

[acel13038-bib-0053] Villeda, S. A. , Plambeck, K. E. , Middeldorp, J. , Castellano, J. M. , Mosher, K. I. , Luo, J. , … Wyss‐Coray, T. (2014). Young blood reverses age‐related impairments in cognitive function and synaptic plasticity in mice. Nature Medicine, 20, 659–663. 10.1038/nm.3569 PMC4224436 24793238

[acel13038-bib-0054] Walker, R. G. , Poggioli, T. , Katsimpardi, L. , Buchanan, S. M. , Oh, J. , Wattrus, S. , … Lee, R. T. (2016). Biochemistry and biology of GDF11 and myostatin: Similarities, differences, and questions for future investigation. Circulation Research, 118(7), 1125–1142; discussion 1142. 27034275 10.1161/CIRCRESAHA.116.308391 PMC4818972

[acel13038-bib-0055] Wei, M. , Brandhorst, S. , Shelehchi, M. , Mirzaei, H. , Cheng, C. W. , Budniak, J. , … Di Biase, S. , et al. (2017). Fasting‐mimicking diet and markers/risk factors for aging, diabetes, cancer, and cardiovascular disease. Science Translational Medicine, 9,(377), 1–12. pii: eaai8700. 10.1126/scitranslmed.aai8700 PMC6816332 28202779

[acel13038-bib-0056] Weindruch, R. , & Sohal, R. S. (1997). Seminars in medicine of the Beth Israel Deaconess Medical Center. Caloric intake and aging. New England Journal of Medicine, 337, 986–994. 10.1056/NEJM199710023371407 9309105 PMC2851235

[acel13038-bib-0057] Wu, H. H. , Ivkovic, S. , Murray, R. C. , Jaramillo, S. , Lyons, K. M. , Johnson, J. E. , & Calof, A. L. (2003). Autoregulation of neurogenesis by GDF11. Neuron, 37, 197–207. 10.1016/S0896-6273(02)01172-8 12546816

[acel13038-bib-0058] Yamamoto, K. , Kiyohara, T. , Murayama, Y. , Kihara, S. , Okamoto, Y. , Funahashi, T. , … Shinomura, Y. (2005). Production of adiponectin, an anti‐inflammatory protein, in mesenteric adipose tissue in Crohn's disease. Gut, 54, 789–796. 10.1136/gut.2004.046516 15888786 PMC1774527

[acel13038-bib-0059] Yamauchi, T. , Kamon, J. , Ito, Y. , Tsuchida, A. , Yokomizo, T. , Kita, S. , … Kadowaki, T. (2003). Cloning of adiponectin receptors that mediate antidiabetic metabolic effects. Nature, 423, 762–769. 10.1038/nature01705 12802337

[acel13038-bib-0060] Yau, S. Y. , Li, A. , Hoo, R. L. , Ching, Y. P. , Christie, B. R. , Lee, T. M. , … So, K. F. (2014). Physical exercise‐induced hippocampal neurogenesis and antidepressant effects are mediated by the adipocyte hormone adiponectin. Proceedings of the National Academy of Sciences of the United States of America, 111, 15810–15815. 10.1073/pnas.1415219111 25331877 PMC4226125

[acel13038-bib-0061] Zhang, R. , Chen, H. Z. , & Liu, D. P. (2015). The Four Layers of Aging. Cell Systems, 1, 180–186. 10.1016/j.cels.2015.09.002 27135911

[acel13038-bib-0062] Zhang, W. , Guo, Y. , Li, B. , Zhang, Q. , Liu, J. H. , Gu, G. J. , … Xu, J. R. (2018). GDF11 Rejuvenates Cerebrovascular Structure and Function in an Animal Model of Alzheimer's Disease. Journal of Alzheimer's Disease, 62, 807–819. 10.3233/JAD-170474 29480172

