# Supplementary material for: Systemic GDF11 stimulates the secretion of adiponectin and induces a calorie restriction‐like phenotype in aged mice
Source: Aging Cell. 2019 Oct 22;19(1):e13038. doi: 10.1111/acel.13038 (PMC6974718; doi:10.1111/acel.13038)
Supplement: Supplementary file 1 [file ACEL-19-e13038-s001.docx]

**Katsimpardi et al. SUPPLEMENTARY INFORMATION**

**Supplementary Materials and Methods**

**Behavioral tests**

Open Field test: The apparatus consists of 4 gray open boxes (arenas), each 50 x 50 cm. Each mouse was placed in the center of the arena, under low light conditions (40lux) to prevent stress, and left to explore freely for 20 minutes. A camera above the arena automatically captured the locomotor activity of the mouse and its behavioral pattern was measured and analyzed using EthoVision XT software (Noldus).

Rotarod: To measure motor coordination, the test was performed in two phases: the training phase and the testing phase, within the same day. During the training phase, mice were placed on a rotating rod at a constant speed of 4 rpm for 2 minutes. This training phase was repeated twice. Mice were placed back into their cages to rest for 2 hours before the test. For the testing phase, mice were placed back onto the rotating rod for 5 min, which accelerated from 4 rpm to 40rpm within 5min (angular acceleration, α=432 rad/s^2^). The latency to fall was measured as the time when the mouse first fell off the rod. The test phase was repeated twice. Measurement of the latency was considered the best out of 3 trials for each mouse.

**Mouse serum collection**

Blood was collected via intracardiac puncture and transferred to EDTA-containing or Microvette 200 Z-Gel (Cat# 20.1344, Fisher Scientific) tubes for serum isolation. To isolate serum, blood was spun down at 1,000*g* for 10min and the supernatant was transferred into new tubes. Serum was snap-frozen and stored at -80 °C.

**Adipocyte cultures**

3T3-L1 cells were purchased from ATCC (ATCC^®^ CL-173^™^) and maintained in T75 flasks in DMEM-10%FBS medium. To initiate adipocyte differentiation 3T3-L1 cells were plated in 6-well plates at a concentration of 0.8x10^5^ cells/well. After cells reached confluence for 2 days, medium was changed to induction medium containing 0.5 mM 3-isobutyl-1-methylxanthine (IBMX), 1μM Dexamethasone and 10μg/ml Insulin in DMEM in 10% Fetal Bovine Serum (FBS) for 2 days. Then medium was changed to 15μg/ml Insulin in DMEM in 10% FBS for the rest of the experiment. Maturation of adipocytes was assessed by the size of lipid droplets viewed by Oil red O staining.

Recombinant GDF11 (Peprotech, Cat# 120-11) and Activin A (Peprotech, Cat# AF-120-14E) were dissolved in H_2_0 and further diluted in 0.1%BSA/PBS to make stocks. Testing of recombinant proteins (20ng/ml) was performed in DMEM/10% FBS medium.

**Western blots**

Cells were lysed in RIPA lysis buffer (25mM Tris-HCl pH 7.6, 150 mM NaCl, 1% NP-40, 1% sodium deoxycholate, 0.1% SDS) (Pierce Thermo Scientific) and protease (cOmplete, Sigma) and phosphatase (phosSTOP, Sigma) inhibitors. Protein concentration was measured with Pierce BCA protein Assay Kit. Tissue lysates were mixed with 4× NuPage LDS loading buffer (Invitrogen), and proteins were separated on a 4–12% SDS-polyacrylamide gradient gel (Invitrogen) and subsequently transferred by semi-dry or liquid transfer onto a PVDF membrane (Trans-blot Turbo Mini PVDF, Biorad). The blots were blocked in 5% BSA in Tris-buffered saline with Tween (TBS-T) and incubated with mouse anti-GDF11 (1:1000, MAB 19581, R&D), rabbit anti-GDF11/8 (1:1000, ab124721, abcam), rabbit anti-adiponectin (1:1000, #2789, Cell Signaling Technology), and mouse anti-actin (1:6000, A5441, Sigma). To detect protein signal the following Horseradish peroxidase–conjugated secondary antibodies were used: Goat Anti-Rabbit IgG (H + L)-HRP Conjugate (1:6000, #1706515, Biorad) and Goat Anti-Mouse IgG1 heavy chain (HRP) (1:6000, ab97240, abcam). Chemiluminescence detection of proteins was performed with Luminata Crescendo Western HRP Substrate (MERCK MILLIPORE) with a Chemidoc Imaging System (Biorad). Bands were quantified using Fiji (ImageJ) software.

-For serum samples, 5 μl of each sample were loaded on the gel.

-For CR plasma, the Proteominer (Bio-rad) enrichment kit was used according to the manufacturer's instructions prior to loading the samples in order to remove the background signal from highly abundant proteins.

-For GDF11 detection in Western blots, we used two antibodies: mouse anti-GDF11 (1:1000, MAB 19581, R&D), which is specific for GDF11 and does not detect GDF8/MST, and rabbit anti-GDF11/8 (1:1000, ab124721, abcam), which gives a stronger signal, to confirm our results. The incubations of the antibodies were performed on different membranes to avoid cross-contamination, and then the blots were stripped and re-probed to cross-confirm the results.

**Anti-GDF11 antibody validation for sensitivity and specificity**

Because of the previous controversy concerning specific detection of GDF11 in the serum, before performing Western blotting for GDF11 in the serum, we first validated antibodies used in previous publications, such as anti-GDF11 (R&D MAB15981- (Egerman et al., 2015) and anti-GDF8/11 (abcam, ab124721) on known concentrations of recombinant GDF11 (Peprotech, Cat# 120-11) and recombinant Myostatin (Peprotech, Cat# 120-00) in separate blots. Increasing concentrations of rGDF11 and rMST were loaded side-by-side in each blot. One blot was incubated with the anti-GDF11 antibody and the other one with anti-GDF8/11, which is known to recognize both GDF11 and MST (Poggioli et al., 2015). The Precision Plus protein Kaleidoscope standards ladder was used (Bio-Rad #1610375). We found that anti-GDF11 shows both specificity and sensitivity in detecting GDF11 as on one hand it detects only the rGDF11 protein and not rMST at any concentration, and on the other hand it shows dose dependency as the intensity increases with increasing concentrations of rGDF11 (Fig. S1A). When we artificially overexposed the same blot MST was still not detected by the anti-GDF11 antibody (Fig. S1B). As previously shown (Poggioli et al., 2015), the anti-GDF8/11 antibody indeed detects both GDF11 and MST, with a higher affinity for MST than for GDF11 (Fig. S1C). This experiment was repeated 5 times with different lots of rGDF11 and the same results were observed each time.

**Sandwich ELISA immunoassays**

The following kits were used for ELISA immunoassays: IGF-1 (22-IG1MS-E01, Alpco), Leptin (EZML-82K, Merck/Millipore) Corticosterone (ADI-900-097, Enzo Life Sciences), GDF15 (MGD150, R&D), insulin (CC-90080, Clinisciences), Ultra sensitive total/HMW adiponectin (47-ADPMS-E01, Alpco), GDF11 (DY1958, R&D; details below). All immunoassays were performed and analyzed according to the manufacturer's instructions.

*Note for GDF11 ELISA*: As a specificity control we used recombinant MST, at the same concentration as the highest GDF11 standard (2ng/ml), as well as at the middle value of the GDF11 standard curve (0.5ng/ml). MST was not detected at any concentration (Fig. 1A), confirming the specificity of this assay for GDF11. This assay was repeated three times and MST was never detected.

**Brain immunostaining**

Mice were anesthetized with a mix of ketamine (80-100 mg/kg) and xylazine (10-12.5 mg/kg) and their brains were removed and fixed overnight in 4% PFA. Each brain was embedded in 4% agarose, and 40μm-thick coronal sections were cut using a vibrating microtome (VT1000S, Leica). Tissue sections or cells were pre-incubated in 10% normal goat or donkey serum, 0.1% Triton-X 100 in PBS for 1h and were incubated overnight at 4 °C with chicken polyclonal anti-doublecortin (DCX) (abcam, ab153668). Alexa Fluor® anti-chicken 647 (Life Technologies) secondary antibody was used for detection of the primary antibody at a dilution of 1:1000 and Hoechst 33342 stained nuclei.

Lipid staining of adipocytes was performed with BODIPY (D3922, Molecular Probes) diluted in PBS at a concentration of 1 mg/mL and applied on fixed adipocytes for 30 mins.

**Muscle sections**

TA muscles were isolated from mice and frozen directly in liquid nitrogen cooled isopentane for < 1 min and stored at 80°C or directly cryosectioned in 10um sections. For histology, TA sections were fixed with 4% PFA in PBS, washed and then routinely stained with Haematoxylin and Eosin (H&E).

**Image acquisition and analysis/quantification**

Muscle imaging was performed using an Olympus CKX41 microscope, a CoolLED pE-300 white lamp, an Olympus U-LS30-3 Camera and CellSens Entry imaging software.

Brain imaging was performed using a Zeiss Apotome microscope. Quantification of DCX surface was performed using the Icy software (<http://icy.bioimageanalysis.org/>).

**Statistical analyses**

All statistical analyses were performed using GraphPad Prism, with one-way or two-way ANOVA and Tukey's *post hoc* test for multiple group comparisons. Mann-Whitney test was used for two-group comparisons, assuming a two-tailed distribution. Statistical significance was assigned for p<0.05; results are shown as standard error of the mean (S.E.M.).

**Supplementary Figures**


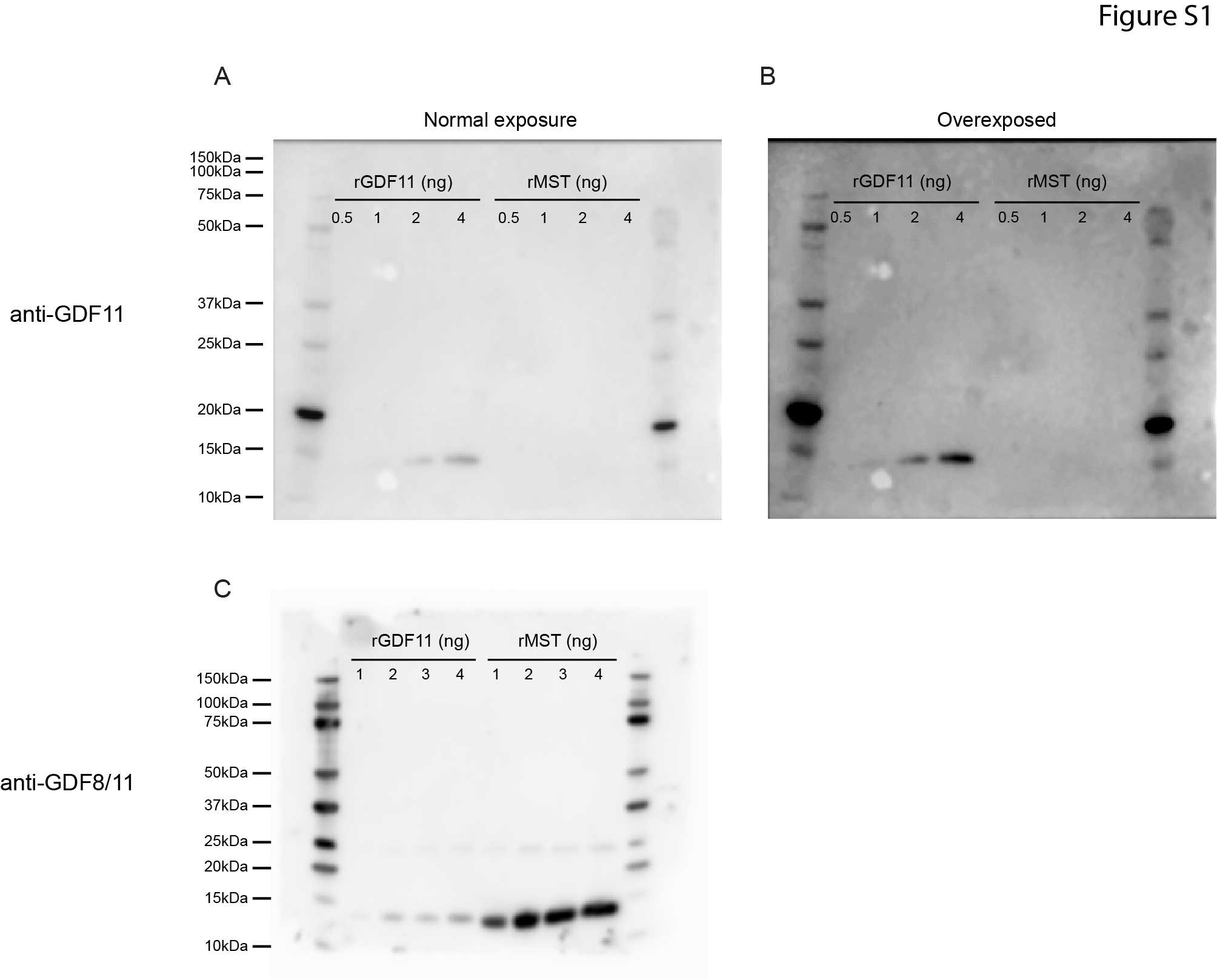


**Supplementary Figure S1. Validation of the specific anti-GDF11 antibody**

**(A)** Representative Western blot image of increasing concentrations of rGDF11 and rMST (0.5, 1, 2, 4ng) probed with the specific anti-GDF11 antibody (see Materials and Methods) showing specificity and sensitivity for GDF11 but not MST. **(B)** Same blot as in (A), overexposed 10 times in order to demonstrate the non-detection of rMST. **(C)** Representative Western blot image of increasing concentrations of rGDF11 and rMST (1, 2, 3, 4ng) probed with the anti-GDF8/11 antibody (see Materials and Methods).


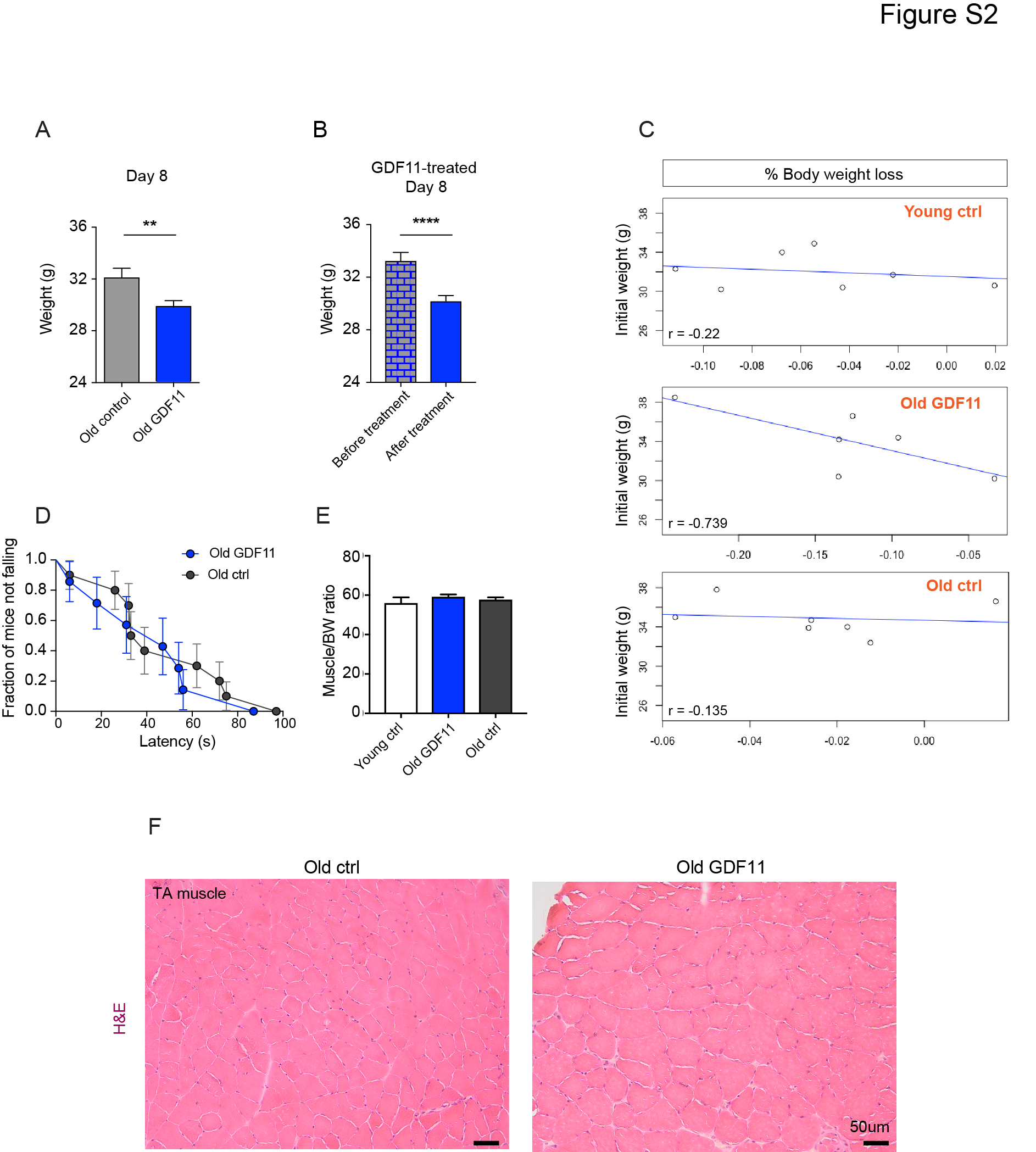


**Supplementary Figure S2. Body weight and muscle analysis after systemic GDF11 treatment**

**(A)** Comparison of body weight between GDF11- or saline-treated mice after the first week of treatment (n_O_=7, n_GDF11_=6 mice per group). **(B)** Comparison of body weight between GDF11-treated mice before and after the first week of treatment (n=16 mice per group). **(C)** Correlation analysis between initial weights of mice and the percentage of body weight loss of the same mice over the course of the experiment. The blue line shows the best-fit linear regression and *r* is Pearson's correlation coefficient. **(D)** Mice were evaluated for their locomotor and coordination performance using the Rotarod test. The survival curve represents the fraction of mice not falling from the Rotarod over the latency to first fall. The data represent the best out of three trials for each mouse. No statistical significance was observed with the non-parametric Kruskal-Wallis test (*P*=0.0371), followed by Dunn's multiple comparisons test. **(E)** Measurement of ratio of tibialis anterior muscle mass over body weight (n_Y_=4, n_GDF11_=10, n_O_=10 mice per group). **(F)** Histological analysis of tibialis anterior muscle sections after a 3-week GDF11 or saline treatment in aged mice. Scale bar: 50μm. One-way ANOVA and Tukey's *post hoc* test for multiple group comparisons. Mann-Whitney test for two-group comparisons; ** *P*<0.01, **** *P*<0.0001; mean ± S.E.M.


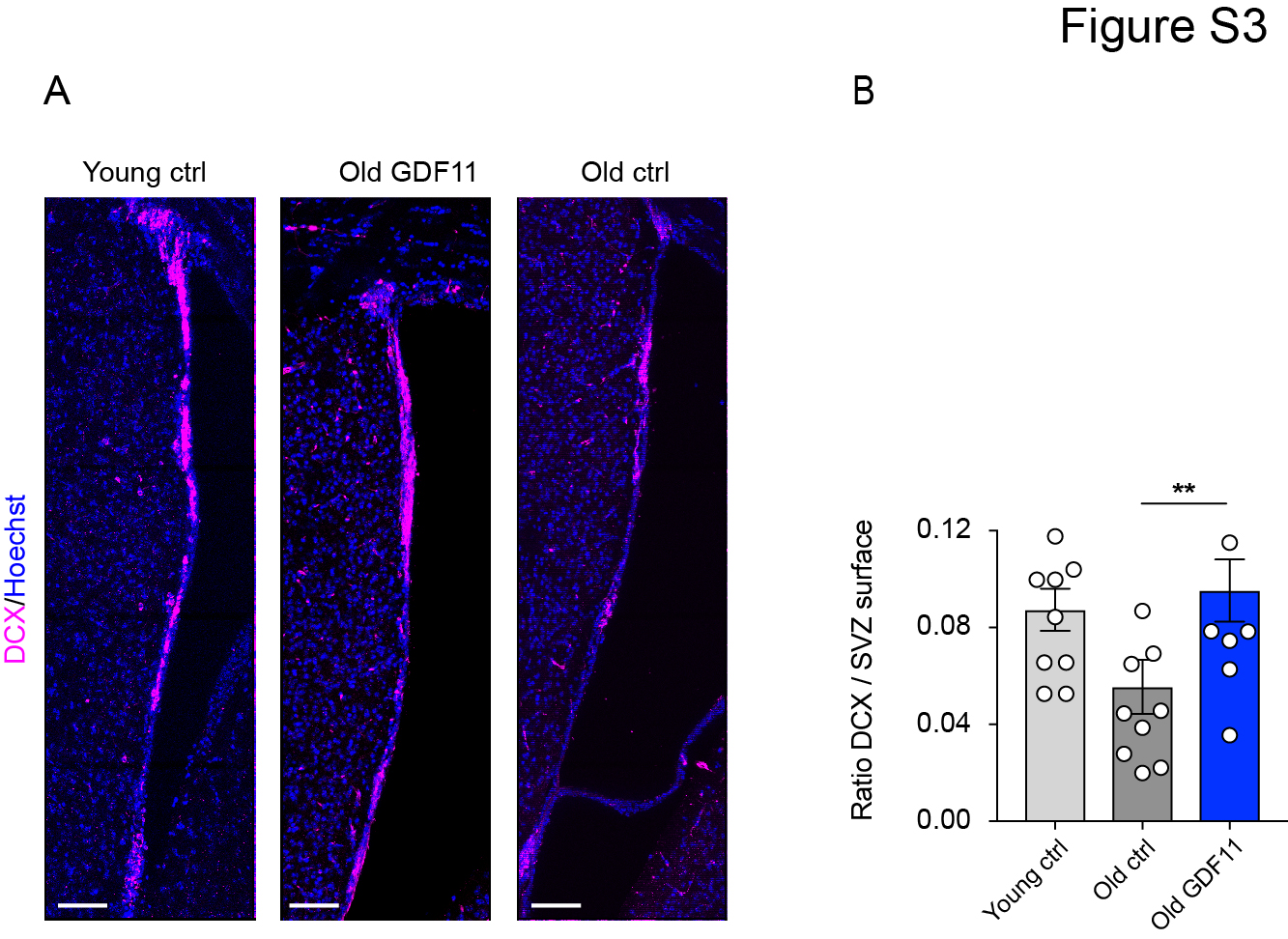


**Supplementary Figure S3. Brain rejuvenation in GDF11-treated aged mice that lost weight**

**(A)** Representative Apotome images of the SVZ immunostained for DCX (purple) and Hoechst (blue) to label neuroblasts and nuclei respectively. Scale bar: 100μm. **(B)** Quantification of the ratio of total DCX intensity over SVZ surface after a 3-week GDF11 or saline treatment in aged mice. (n_Y_=9, n_GDF11_= 6, n_O_= 0 mice per group). One-way ANOVA and Tukey's *post hoc* test for multiple group comparisons; ** *P*<0.01, mean ± S.E.M.

**
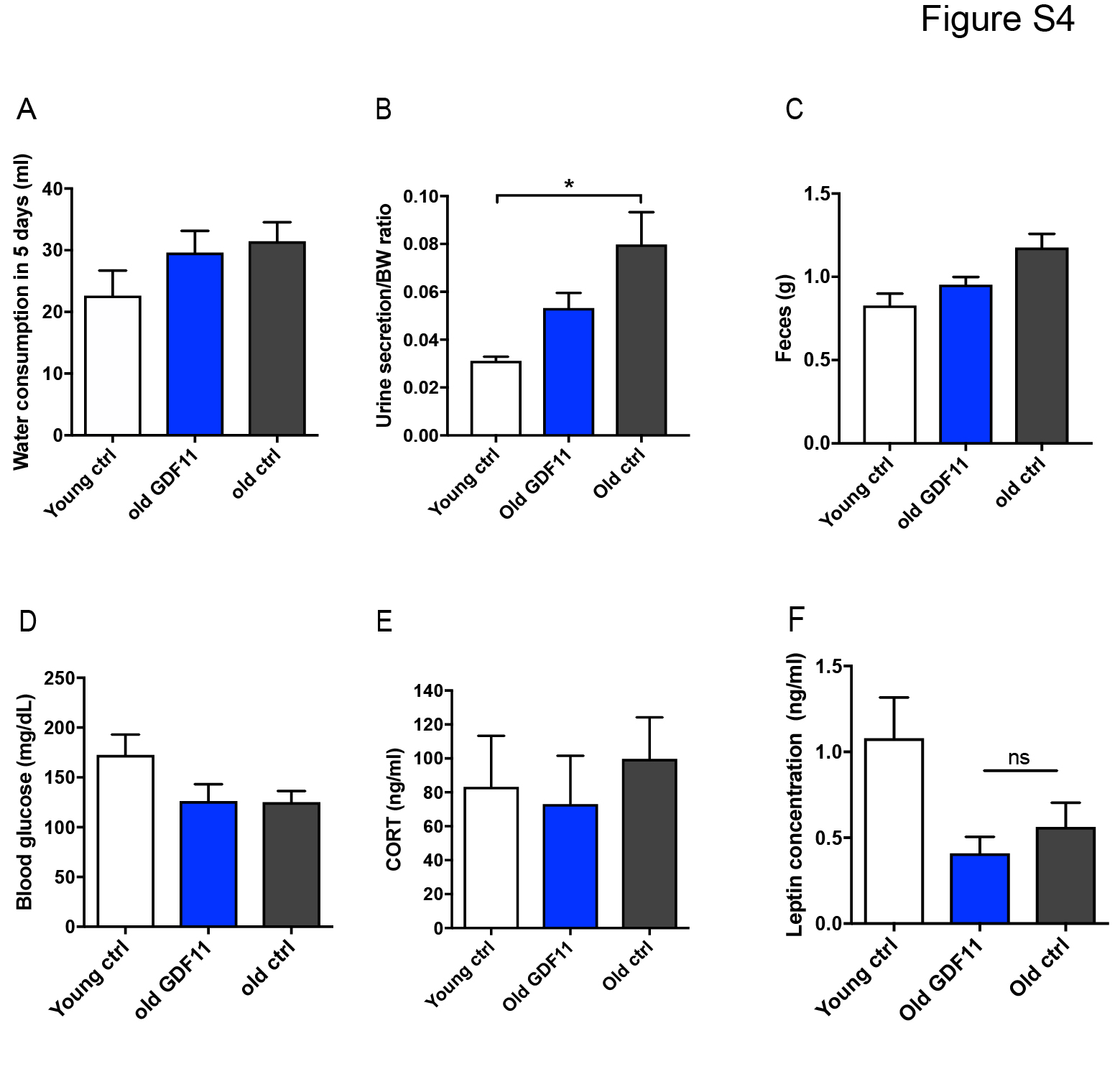
**

**Supplementary Figure S4.** **Physiological parameters measured in metabolic cage experiments**

**(A)** Measurement of total water consumption in 5 days (n_Y_=4, n_GDF11_=10, n_O_=10 mice per group). **(B)** Measurement of average urine secretion per day (n_Y_=4, n_GDF11_=10, n_O_=10 mice per group). **(C)** Measurement of average feces secretion per day (n_Y_=4, n_GDF11_=10, n_O_=10 mice per group). **(D)** Levels of blood glucose after 12h fasting (n_Y_=8, n_GDF_=9, n_O_=12 mice per group). **(E)** ELISA measurement of corticosterone levels at the end of the experiment (n_Y_=2, n_GDF_=5, n_O_=5 mice per group). **(F)** ELISA measurements of circulating leptin in plasma of young, old and GDF11-treated old mice (n_Y_=8, n_GDF11_=10, n_O_=10 mice per group). One-way ANOVA and Tukey's *post hoc* test for multiple group comparisons; **P*<0.05; mean ± S.E.M.
